# The Chinese version of attitudes towards guidelines scale: validity and reliability assessment

**DOI:** 10.1186/s12874-019-0682-3

**Published:** 2019-02-22

**Authors:** Wen Yan, Qian Chen, Xuemei Zhang, Marko Elovainio, Yan Huang

**Affiliations:** 1 State Key Laboratory of Oral Diseases & National Clinical Research Center for Oral Diseases & Other Research Institutes & Department of Nursing, West China Hospital of Stomatology, Sichuan University, ChengDu city, China; 2 Department of Geriatrics Center, National Clinical Research Center of Geriatrics, West China Hospital, Sichuan University, ChengDu city, China; 3 National Institute for Health and Welfare (THL), Helsinki, Finland; 4 University of Helsinki, Helsinki, Finland

**Keywords:** Psychometric properties, Survey, Clinical, Quality, Guidelines

## Abstract

**Background:**

The use of guidelines has shown to improve clinical practice process and structure of health care, but health care providers don’t always use and keep up-to-date with the new clinical practice guidelines. Nurses’ attitudes towards guidelines have shown to be the most frequently identified factor affecting their actual use of clinical practice guidelines, but no instruments for measuring it are available in China. There are scales validated in the western countries, but there is no information about their validity in Chinese health care. The purpose of this study is to test the validity and reliability of Chinese Attitudes towards guidelines - scale for nurses.

**Methods:**

The study was conducted from April to July 2017. The Attitudes towards guidelines scale was translated into Chinese with forward-backward translation method and a questionnaire survey was conducted. Eight hundred randomly selected nurses (final *N* = 768) from Geriatrics, Internal medical and Rehabilitation departments of 16 hospitals were drawn in Sichuan province, China. Construct validity was evaluated by exploratory and confirmatory factor analysis, and reliability was assessed by test-retest reliability (represented by intra class correlation) and internal consistency (expressed by Cronbach’s coefficients). The test-retest reliability was examined with a sample of 32 clinical nurses who filled out the questionnaire 14 days after the first survey.

**Results:**

Exploratory factor analysis supported a four-factor model for the Chinese version of the scale. Confirmatory factor analysis indicated that the hypothetical four-factor model fitted the data relatively well. The intra class correlation coefficient was 0.85 (95%CI, 0.68–0.93) and the Cronbach’s alpha values for the four subscales ranged from 0.645 to 0.912.

**Conclusions:**

The results support the acceptable level of validity and reliability of the Chinese version of Attitudes towards guidelines scale, which can be used to assess nurses’ attitudes towards guidelines in China. Future testing for the Chinese version of Attitudes towards guidelines scale needs to be carried out to see whether these results are generalizable to other professionals and occupational groups and to be used to revise attitudes towards specific guidelines in China.

## Background

Clinical practice guidelines (abbreviated as guidelines) are evidence-based tools for standardized treatment programs which can bridge the gap between science and practice and help health care providers conduct evidence-based clinical decisions [1]. The use of guidelines has been shown to improve clinical practice process and structure of health care [2]. Potential benefits of using guidelines on clinical practice include improving patients’ health outcome such as reducing morbidity and mortality, improving the quality of their life, providing consistency of care [3], and reducing health care costs [4].

However, health care providers don’t always use and keep up-to-date with the new clinical practice guidelines. Researches have shown that no more than 40% of health workers followed the guidelines [5] and less than 25% of guidelines were used by clinicians [3]. Research also shows nurses have poor knowledge of their professional guidelines [6]. Since nurses are the largest occupational group of health care providers, many studies have done to explore the factors that affect nurses’ use of guidelines [6–12]. An integrative review of 16 related articles examining the barriers and facilitators of nurses’ use of clinical practice concludes that the most frequently identified barriers and facilitators of nurses’ use of guidelines are their attitudes and perceptions towards guidelines [13]. Those who have more positive attitudes towards guidelines more often use guidelines, while their negative attitudes towards guidelines are related to a decreased use of guidelines [7, 13]. Chinese study [14] also shows that two of the obstacles for nurses’ use of guidelines are questioning the evidence and fearing that the guidelines would be bad for patients. But there is a lack of validated instruments measuring the attitudes towards guidelines that is suitable for Chinese health care system, although there are more than 3.5 million registered nurses in China.

Potentially suitable instruments have been developed in the western countries. Of them probably the most widely used is the Attitudes towards Guidelines - scale (AGS) developed in a multi-national project [4]. AGS was designed to evaluate health care professionals’ attitudes towards guidelines in multiple health care systems and the English, Finnish and Spanish version of the AGS have shown to be a valid and reliable tool for assessing the attitudes towards guidelines in multiple western health care systems. The purpose of this study is to test whether the AGS could be used in China by looking at the psychometric properties. And thus it would be a useful tool for measuring attitudes towards guidelines in China. The practical implications of detecting the factors affecting the use of clinical guidelines among Chinese nurses would be large considering the scale of the Chinese population.

## Methods

### Design

A cross-sectional and descriptive design was used in this study.

### Study sample

A stratified random sampling method was used in Secondary-level and Tertiary-level Hospitals in Sichuan province of China from April to July 2017. First, according to geographical location, the researchers divided Sichuan province into four regions: East, South, West and North. Then, a random number table was used to select two secondary-level and two tertiary-level hospitals in each region that agreed to participate in this study. A total of 16 hospitals were selected to participate in this study. Finally, 50 nurses working in the Department of Internal Medicine, Rehabilitation and Geriatrics in each hospital were selected randomly with a random number table method. A total of 800 nurses participated in the study and of them 768 provided valid questionnaires and formed the final sample.

In addition to a cover letter that expressed the purpose and importance of the research, the survey questionnaire that needed nurses to complete consisted of three parts: the first part was an personal information form which was used to determine the characteristics of participants, and the second part was the Chinese Version of AGS and as this study was conducted concurrently with another study on the application of the fall guideline for the elderly, so the third part was an elderly fall guideline knowledge questionnaire. The beginning of each part was a detailed instruction. The researchers went to each hospital to conduct the survey. Nurses who had questions could ask the researcher in that hospital. Each participant needed 15–20 min to complete the whole questionnaire.

### The attitudes towards guidelines -scale

The attitudes towards guidelines scale consists of 14 items that are categorized into seven subscales (two items each): (1) general attitude towards guidelines, (2) usefulness of guidelines, (3) reliability of guidelines, (4) lack of individual or team competence, (5) lack of organizational competence, (6) impracticality of guidelines, (7) availability of guidelines. Responses to the items are scored on a 7-point scale ranging from strong disagree 1 to strong agree 7. The scoring of subscales (4), (5) and (6) are reversed. The total score is ranging from 14 to 98. The higher the score, the more positive the attitude.

After getting the permission of the authors to use the AGS, we followed the forward-backward translation method [15] to translate the scale from English to Chinese. Two bilingual nursing graduate students translated the scale into Chinese and formed two translation versions. A bilingual nursing expert synthesized the two versions into back translation version, and another two bilingual language graduate students who had no knowledge of the original version back-translated the scale into Chinese independently. Finally, an expert committee consisting of one methodologist, all translators and researchers reviewed all translation and cultural adaptation processes. Because we wanted to investigate nurses’ attitudes towards guidelines, the wording in the eleventh item was changed from “care providers” to “nursing personnel”, so that the item read: “Guidelines challenge the autonomy of nursing personnel”. And the statement of the twelfth item was changed from “medical practice” to “nursing practice”, so that the item was expressed as: “Guidelines oversimplify nursing practice”. According to the Chinese habits, the response to the items was changed to a 5 points, ranging from strongly disagree 1 to strongly agree 5. When consensus was reached, a pre-final version of the scale was obtained. To reach a final version of the scale, pretesting was carried out with 35 nurses who were hospital clinical nurses. The purpose was to ensure the Chinese version of the scale could be understood and to make sure the items measured the contents they wanted to measure. The nurses were encouraged to ask and discuss any seemingly confusing item. All nurses said they could understand the scale and the researchers assessed the results and formed the final Chinese version.

### Content validity

Content validity means the degree to which the items of an instrument fully reflect the structure to be tested [16]. The content validity of the study was evaluated using the item-level content validity index (I-CVI) [17]. A panel of six experts in nursing management and clinical care (three in each group) assessed the relevance of the initial 14 items to the nurses’ attitudes towards guidelines, using the following 4-point scale: 1 = “not relevant”, 2 = “somewhat relevant”, 3 = “quite relevant”, and 4 = “very relevant”. The I-CVI is quantified as the ratio of experts who answer the item “quite relevant” or “very relevant”. Items with I-CVI values greater than 0.78 are considered sufficiently relevant to the construction of nurses’ attitudes towards guidelines [17]. The 14 items were all with I-CVI values > 0.78, ranging from 0.83 to 1.0, so no items were deleted.

### Data analysis

Data analyses were performed using SPSS 19.0 and Mplus 7.4 software. The demographic characteristics of subjects were analyzed by using descriptive statistical method. Data were represented by means (with SDs) or numbers (with percentages).

For the cross-validation of factorial construct validity, the total sample was divided into two groups (group A and group B) randomly by SPSS 19.0. The sample size of group A was 380, and that of group B was 388. Group A was used for exploratory factor analysis (EFA) and group B was used for confirmatory factor analysis (CFA). Both EFA and CFA were carried out with Mplus 7.4 software, using a weighted least squared means and variance adjusted (WLSMV) estimator method that was designed for ordinal data [18]. EFA was performed using a geomin oblique rotation. Item loading>0.45 in the factor is considered acceptable [19]. CFA model fit was evaluated using the comparative fit index (CFI) [20], the Tucker-Lewis index (TLI) [21], the standardized root mean square residual (SRMR) [22] and the root mean square error of approximation (RMSEA) [23]. For CFI and TLI, values between 0.90 and 0.95 are considered acceptable, and ≥ 0.95 are good [22]. Values of SRMR≤0.08 [22] and values of RMSEA≤0.1 [23] are considered acceptable. The reliability of the Chinese version of AGS and its subscales was measured using test-retest reliability and internal consistency. The test-retest reliability was measured using the intra-class correlation coefficient (ICC) and its 95% confident interval which was calculated using SPSS statistical package version 19 based on a single-rating, absolute-agreement, 2-way mixed-effects model [24] with a sample of 32 clinical nurses selected conveniently who filled out the questionnaire at an interval of 14 days [25]. ICC values between 0.5 and 0.75 indicate moderate. Values between 0.75 and 0.9 showe good reliability, and values greater than 0.90 indicate excellent reliability [25].The internal consistency of a scale was measured by the Cronbach’s a coefficient (α) that ranged from 0 to 1, which was acceptable at α = 0.60–0.69, good at α = 0.70–0.89, and excellent at α = 0.9–1.0 [26].

### Ethical considerations

The study was approved by the Ethics Committee of West China Hospital of Sichuan University (reference number: 201772). The research’s purpose and benefits were explained to the nurses, and written consent to participate in the research was obtained on a voluntary basis. The nurses completed the questionnaires without signature.

## Results

### The sample

Among the 800 nurses, the participant rate was 96% (768/800), of which 2.7% were male and 97.3% were female. The average age was (28.23 ± 6.37) years old. More than half of nurses were married (56.9%). The majority of participants were Bachelor degree (44.5%). Half of the nurses’ working years were among 1–5 years (51.9%). There were an almost equal proportion of participants who were working in Tertiary-level hospital (49.9%) and Secondary-level hospital (50.1%). The majority of participants were from Internal medical departments (59.7%) and Geriatrics wards (28.3%). Most of the nurses were general nurse (80.1%). More than three-quarters of the participants didn’t learn guidelines of their fields in the past six months before the survey (82.7%). The AGS score ranged from 26 to 70 with a mean score of 48.12 (standard deviation [SD] =6.08).

### Construct validity

#### CFA with the whole sample

We conducted confirmatory factor analysis according to the conceptual structure of the original version of the AGS with the whole data (768 subjects). As shown in Table 1, the model fit for the original 7-factor structure (model 1) was poor.

**Table 1:** CFA Fit Indices for the 7-Factor Model for nurses’ attitudes towards guidelines (*n* = 768)

Model ^a^	χ^2 b^	DF ^b^	CFI ^b^	TLI ^b^	SRMR ^b^	RMSEA ^b^	95%CI
Model 1	422.618	56	0.930	0.887	0.094	0.092	0.084–0.101

^a^Model 1: according to the conceptual structure of the original version of the AGS

^b^χ^2^ = chi-square; *DF* Degrees of freedom, *CFI* Comparative fit index, *TLI* Tucker-Lewis index, *SRMR* Standardized root mean square residual, *RMSEA* Root mean square error of approximation

#### EFA with group a and the second CFA with group b

Since the original factor structure didn’t fit the data well, we conducted exploratory factor analyze with the data of group A to check the number of factors extracted and their related items. Seven alternative models of the scale were estimated in Mplus 7.4 software, which were from1-factor model to 7-factor model respectively. The result showed that two models (4-factor and 5-factor model) had adequate fit indices (Table 2). However, there were 3 variable loadings≤0.45 in 4-factor model (Table 3), while the content of items in two factors of the 5-factor structure could not be summarized as a meaning. Then we went through the items of the AGS again to determine if a different list of items could be used to meaningfully capture the Chinese nurses’ attitudes towards guidelines. We found that we couldn’t improve the 5-factor structure. But after deleting three items loadings≤0.45 (Item 9,11 and 12) of the 4-factor structure, we found that 4-factor structure (11 items) could be well explained: (1) usefulness of guidelines (Item 1,2,3 and 4), (2) reliability of guidelines (Item 5 and 6), (3) lack of individual or team competence (Item 7 and 8), (4) availability of guidelines (Item 10,13 and 14) (Table 3). Then we conducted the second CFA with the 4-factor structure (11 items) which we named model 2 in this study using the data of group B. The result showed that the 4-factor solution in CFA was acceptable (RMSEA = 0.083, CFI = 0.957, TLI = 0.938 and SRMR = 0.036), and this improved after including the two most significant error covariances, between item 1 and 2, 13 and 14. After modifications, the values in Model 2 indicated an excellent fit with the observed data (Table 4).

**Table 2:** Fit indices for seven alternative models of Chinese Attitudes towards guidelines–scale (*n* = 380)

models	χ^2^	DF	CFI	TLI	RMSEA (90% CI)
1-factor	968.222	77	0.641	0.576	0.175 (0.165–0.184)
2-factor	349.652	64	0.885	0.836	0.108 (0.097–0.120)
3-factor	199.597	52	0.941	0.896	0.086 (0.074–0.099)
4-factor	117.201	41	0.969	0.932	0.070 (0.055–0.085)
5-factor	47.748	31	0.993	0.980	0.038 (0.013–0.058)
6-factor	18.073	22	1.000	1.007	0.000 (0.000–0.034)
7-factor	4.690	14	1.000	1.024	0.000

χ^2^ = chi-square; *DF* Degrees of freedom, *CFI* Comparative fit index, *TLI* Tucker-Lewis index, *RMSEA* Root mean square error of approximation

**Table 3:** Geomin Rotated Factor Loadings for the Four-Factor Model Derived by EFA with the data of group A (*n* = 380)

Items	Factor1	Factor2	Factor3	Factor4
1. Guidelines are useful as educational tools.	0.910*	− 0.056	− 0.037	0.014
2. Guidelines are a convenient source of advice.	0.940*	0.003	0.007	−0.022
3. Guidelines can facilitate communication with patients and families.	0.734*	0.176*	0.040	−0.060
4. Guidelines can improve the quality of health care.	0.585*	0.368*	−0.011	0.026
5. Guidelines are based on scientific evidence.	0.161	0.766*	0.010	0.000
6. Guidelines are made by experts.	−0.049	0.744*	−0.004	− 0.015
7. My occupational competence is insufficient for adopting the latest guidelines.	0.026	0.038	0.767*	−0.028
8. Most of our team members have disapproving attitudes about guidelines.	−0.106	−0.058	0.697*	0.074
9. Guidelines are not valued in our organization.	0.272*	0.217*	−0.114	0.206*
10. Implementing guidelines is too expensive for us.	0.018	0.016	0.348*	0.489*
11. Guidelines challenge the autonomy of nursing personnel.	0.088	0.004	0.245*	0.071
12. Guidelines oversimplify nursing practice.	0.047	0.131	0.124	0.291*
13. Guidelines are difficult to find if needed.	−0.011	−0.092	−0.011	0.850*
14. I have not seen any guidelines in our health care unit.	−0.075	0.041	0.098	0.599*

*EFA* Exploratory factor analysis; * significant at 5% level

**Table 4:** CFA Fit Indices for the Four-Factor Model for nurses’ attitudes towards guidelines (*n* = 388)

Model ^a^	χ^2 b^	DF ^b^	CFI ^b^	TLI ^b^	SRMR ^b^	RMSEA ^b^	95%CI
Model 2	79.061	36	0.987	0.972	0.035	0.056	0.039–0.072

^a^Model 2: Items selected to load on CFA factors are based on the EFA with the data of group A loading with a cut-off of 0.45

^b^χ^2^ = chi-square; *DF* Degrees of freedom, *CFI* Comparative fit index, *TLI* Tucker-Lewis index, *SRMR* Standardized root mean square residual, *RMSEA* Root mean square error of approximation

#### Reliability

The intra-class correlation coefficients was 0.85 (95%CI: 0.68–0.93) in a sample of 32 hospital clinical nurses and the Cronbach’s alpha values for the four factors of the Chinese version of AGS were 0.912, 0.645, 0.781 and 0.804 respectively. These results indicated that the Chinese version of AGS exhibited acceptable test–retest reliability and internal consistency.

## Discussion

The purpose of this study is to test the validity and reliability of Chinese Attitudes towards guidelines - scale for nurses. The results suggested that the Chinese AGS provided acceptable psychometric properties and worked well in a Chinese context for assessing the nurses’ attitudes towards clinical guidelines. In China, nursing staff mainly carry out nursing operations and nursing measures according to nursing textbooks, but it is well known that the knowledge in the textbooks is relatively backward, and some contents may be not suitable for clinical use. It has been 20 years since evidence-based medicine was introduced into China. With the development of evidence-based medicine in China, guidelines are being gradually understood by Chinese nurses [6]. There is guideline developed by Chinese nurses for standardized clinical practice [27]. However, the results of the survey [6, 14] showed that nurses in China didn’t have a good grasp of the relevant professional guidelines. One of the reasons was that they had some doubts about the guidelines. So the results of this study are meaningful to the Chinese clinical nurses. There are also other tools for measuring the health care providers’ attitudes towards guidelines [7, 28], but they are developed for a particular guideline such as the hand hygiene guideline and the item number of the scale exceeds that of the AGS. Since the Chinese AGS has acceptable validity and reliability, it can be revised in the future to measure Chinese nurses’ attitudes towards specific guideline such as elderly fall guideline.

Cross-cultural adaption was the key step to introduce AGS into China. The quality of cross-cultural adjustment of scale will affect its psychological measurement index. The cross-cultural adaptation procedure [7, 28] we followed in the study was standardized and commonly used in the world. In addition, we closely contacted with the original authors [29] and sent the translated, synthesized, and back-translation versions to the original authors via e-mail, inviting them to evaluate the differences between the original version and the Chinese version of the scale. Through communication with the original author, we further understand the purpose and significance of the original author’s development of the scale. The whole process was scientific and complete, guaranteeing the equivalence between the content of the Chinese version of AGS and the source scale.

Findings from our study showed that the data didn’t fit to original version of seven-factor model [29] (model 1), while model 2 based on the EFA results with four well explained factors loading with a cut-off of 0.45 indicated an acceptable fit with the observed data after modifications. One explanation could be the different sample population in the two studies. The population tested in the original version of the attitudes towards guidelines [4] was the health care providers including physicians, dentists, registered nurses and practical nurses, while the sample in the Chinese version tested was registered nurses only. Further testing of the scale with other health care professionals and occupational groups in China is needed to assess the structural validity and to see whether these results are applicable to them. Besides, when we made the cross-cultural adaptation of the scale, we changed the responses to the item from 7 points to 5 points according to the Chinese habit. This may be one of the reasons that lead to structural differences. Four factors obtained from the EFA are: Factor 1 identifies the usefulness of guidelines; Factor 2 is concerned about reliability of guidelines; Factor 3 is related to lack of individual or team competence; Factor 4 is with regard to availability of guidelines. Three deleted items are about a lack of organizational competence and impracticality of guidelines. The reasons for the low loading of these three items may be that most of the samples in the study are relatively young and have little work experience, so that they may less use evidence-based medicine in their work and lack of in-depth understanding of evidence-based medicine. The French version of AGS showed that their research didn’t obtain a suitable model [30]. This may have resulted from cultural influence in the experience of properties and the version of AGS they translated into French was the initial scale of 27 items rather than the final version of 14 items.

The test-retest reliability of the Chinese version of the scale was good with the intra class correlation coefficients above 0.80, and the internal consistency reliability of the Chinese AGS was acceptable with Cronbach’s alpha values of the four factors all above 0.60, which indicating that the scale was reliable.

The limitations of this study are as follows. The present validation study of the Chinese version of the scale was carried out only among clinical nurses from three parts in Sichuan province’s hospitals: Internal Medicine wards, Rehabilitation wards and Geriatrics wards for the whole research purpose. Further investigation is needed to be done in larger populations and more wards to test if the findings are consistent over time, across different wards in different hospitals different provinces in China on nurses’ attitudes towards guidelines. And future testing for the Chinese version of the scale needs to be carried out to see whether it is generalizable to other professionals and occupational groups in China. Another limitation of the present study is the predictive validity of the scale which may make the scale more useful isn’t assessed. It should be undertook in future research. Lastly, the scale need to be further applied to analyze the relationship between the demographic characteristics of nurses and their attitudes towards to the guidelines.

## Conclusion

The four-factor structure (11 items) of the Chinese version of the AGS seems to be a valid and reliable instrument for measuring the nurses’ attitudes towards to guideline in China. The scores of each subscale are ranging from 4 to 20, 2 to 10, 2 to 10 and 3 to 15 respectively. As more and more clinical guidlines are generated and applied to clinical practice [31], it could assist Chinese nursing administrators in optimizing management strategies for clinical guidelines implementation. Due to the size of health care in China, revealing the most important factors that prevent nurses from following the recommendations of clinical guidelines may have a significant impact on patient outcomes.

## Abbreviations

AGS: Attitudes towards Guidelines Scale
CFA: Confirmatory factor analysis
CFI: Comparative fit index
DF: Degrees of freedom
EFA: Exploratory factor analysis
ICC: intra-class correlation coefficient
I-CVI: Item-level Content Validity Index
RMSEA: Root mean square error of approximation
SD: Standard deviation
SRMR: Standardized root mean square residual
TLI: Tucker-Lewis index
WLSMV: Weighted least squared means and variance adjusted
